# Green Synthesis of Silver Nanoparticles with Roasted Green Tea: Applications in Alginate–Gelatin Hydrogels for Bone Regeneration

**DOI:** 10.3390/gels10110706

**Published:** 2024-10-31

**Authors:** Patricia Alejandra Chavez-Granados, Rene Garcia-Contreras, Cesar A. S. Reyes-Lopez, Jose Correa-Basurto, Irving E. Hernandez-Rojas, Gabriela Hernandez-Gomez, Carlos Alberto Jurado, Abdulaziz Alhotan

**Affiliations:** 1 Interdisciplinary Research Laboratory, Nanostructures, and Biomaterials Area, National School of Higher Studies (ENES) Leon, National Autonomous University of Mexico (UNAM), Leon 37684, Mexico; 2 Dental Science, Master’s and Doctoral Program in Medical, Dental, and Health Sciences, National Autonomous University of Mexico (UNAM), Coyoacán, Mexico City 04510, Mexico; 3 National Polytechnic Institute, National School of Medicine and Homeopathy, Mexico City 07320, Mexico; 4 Laboratory of Design and Development of New Drugs and Biotechnological Innovation, SEPI-Escuela Superior de Medicina, National Polytechnic Institute, Plan de San Luis and Díaz Mirón, Mexico City 11340, Mexico; 5 Periodontics and Implantology Area, National School of Higher Studies (ENES) Leon, National Autonomous University of Mexico (UNAM), Leon 37684, Mexicoghernandezg@enes.unam.mx (G.H.-G.); 6 Operative Dentistry Division, Department of General Dentistry, College of Dentistry, University of Tennessee Health Science Center, Memphis, TN 38163, USA; 7 Department of Dental Health, College of Applied Medical Sciences, King Saud University, P.O. Box 10219, Riyadh 12372, Saudi Arabia

**Keywords:** roasted green tea, silver nanoparticles, hydrogel, gelatin, alginate, bone regeneration

## Abstract

The incorporation of silver nanoparticles (AgNPs) into alginate–gelatin (Alg-Gel) hydrogels can enhance the properties of these materials for bone regeneration applications, due to the antimicrobial properties of AgNPs and non-cytotoxic concentrations, osteoinductive properties, and regulation of stem cell proliferation and differentiation. Here, the hydrogel formulation included 2% (*w*/*v*) sodium alginate, 4 µg/mL AgNPs, and 2.5% (*w*/*v*) gelatin. AgNPs were synthesized using a 2% (*w*/*v*) aqueous extract of roasted green tea with silver nitrate. The aqueous extract of roasted green tea for AgNP synthesis was characterized using HPLC and UHPLC-ESI-QTOF-MS/MS, and antioxidant capacity was measured in Trolox equivalents (TE) from 4 to 20 nmol/well concentrations. Stem cells from human exfoliated deciduous tooth cells were used for differentiation assays including positive (SHEDs/hydrogel with AgNPs) and negative controls (hydrogel without AgNPs). FTIR was used for hydrogel chemical characterization. Statistical analysis (*p* < 0.05, ANOVA) confirmed significant findings. Roasted green tea extract contained caffeine (most abundant), (−)-Gallocatechin, gallic acid, and various catechins. XRD analysis revealed FCC structure, TEM showed quasispheroidal AgNPs (19.85 ± 3 nm), and UV–Vis indicated a plasmon surface of 418 nm. This integration of nanotechnology and biomaterials shows promise for addressing bone tissue loss in clinical and surgical settings.

## 1. Introduction

Bone regeneration is a complex physiological process that occurs during the normal healing phase of various injuries, such as fractures [1], periodontal disease [2], cysts, tumors, etc., as well as in continuous remodeling throughout the human lifespan. The treatment of these conditions often involves autologous bone grafts, which have been established as the “gold standard” for bone repair materials due to their properties of osteoconduction, osteogenesis, and osteoinduction [3]. Tissue engineering (TE) is emerging as a strategy that could address the limitations of autografts and allografts [4]. These biomaterials can deliver mechanical and biochemical stimuli that promote the differentiation of stem cells (SCs) into various lineages [5]. The self-renewal and differentiation characteristics of SCs make them an excellent choice for study in tissue engineering and regenerative medicine. One study evaluated the use of adult SCs derived from adipose tissue, which can differentiate into chondrocytes or, if the biomaterial is stiffer, into cardiomyocytes, for example [6]. The processes of osteogenesis and angiogenesis in bone marrow-derived mesenchymal stem cells (bMSCs) can be upregulated with silica nanoparticles [7]. On the other hand, SHED cells (stem cells from human exfoliated deciduous teeth) are of great relevance in differentiation assays [8] as they can differentiate into osteoblasts, chondrocytes, adipocytes, and neurons. Among the biomaterials used in tissue engineering, natural hydrogels made from polymers are highly biocompatible and have physical–mechanical, biological, and chemical properties that resemble the extracellular matrix (ECM) [9]. They also have good permeability to nutrients, oxygen molecules, and other water-soluble materials, with the physical-mechanical properties of the hydrogel determining SC fate [5].

Alginate (Alg) and gelatin (Gel) hydrogels are promising biomaterials for tissue engineering applications due to their biocompatibility and their ability to promote cellular adhesion and differentiation [10]. Alginate is a polysaccharide composed of α-L-Guluronate (G unit) and β-D-Manuronate (M unit), linked by β-1,4-glycosidic bonds. It has been widely used in the fabrication of hydrogels due to its biocompatibility, low cost, and non-immunogenicity and has been approved by the FDA [10,11]. Gelatin is a denatured collagen derived from bovine, porcine, fish, or tissues and is also FDA-approved due to its non-toxicity and good degradability. Gelatin plays a crucial role in enhancing cell adhesion, proliferation, and differentiation, making it an ideal scaffold material for tissue engineering [12].

The combination of alginate and porcine gelatin can provide an appropriate microenvironment for cell proliferation, adhesion, migration, and differentiation [13]. The ionic interaction between sodium alginate and gelatin is driven by the presence of ionizable amino and carboxyl groups, along with hydrogen bonding between the amine and carboxyl groups [11]. These biomaterials are being used as carriers or encapsulates for nanomaterials, combining the benefits of biomaterials with the advantages of nanotechnology. Nanotechnology, from which nanomedicine is derived, involves the use of metallic nanoparticles (NPs) for therapeutic and diagnostic purposes [14]. The production of metallic NPs can be achieved through chemical reactions or physical methods [15]. However, many toxic components are absorbed onto the surfaces of NPs, making them incompatible for biomedical applications [16]. These techniques are being replaced by natural substances, leading to what is known as green synthesis. This method involves the use of environmentally friendly sources, such as microorganisms (fungi, bacteria, and algae) or plant extracts [17]. The green synthesis of silver nanoparticles (AgNPs) using green tea (GT) is possible due to the phenols and flavonoids present, which aid in the formation and stabilization of metallic NPs. AgNPs can be synthesized through a two-step process, beginning with the reduction of Ag^+^ ions to metallic Ag^0^, followed by agglomeration and stabilization, resulting in the formation of colloidal AgNPs [18]. The incorporation of AgNPs into Alg-Gel hydrogels can enhance the properties of these materials for bone regeneration applications, due to the antimicrobial properties of the AgNPs. Although biomaterials show adequate mechanical properties, microbial infections can cause complications and treatment failure. AgNPs at non-cytotoxic concentrations have been shown to have osteoinductive properties, regulating the proliferation and differentiation of mesenchymal stem cells involved in bone regeneration [11].

The aim of the present study was to characterize the aqueous extract of roasted green tea (RGT) used for the synthesis of AgNPs and evaluate the effectiveness of these NPs in combination with an alginate–gelatin hydrogel in promoting the differentiation of stem cells from human exfoliated deciduous teeth (SHEDs) towards the osteogenic lineage, with the goal of future applications in the field of bone regeneration engineering.

## 2. Results and Discussion

### 2.1. Identification of Metabolites and Quantification by HPLC

**Figure 1:**
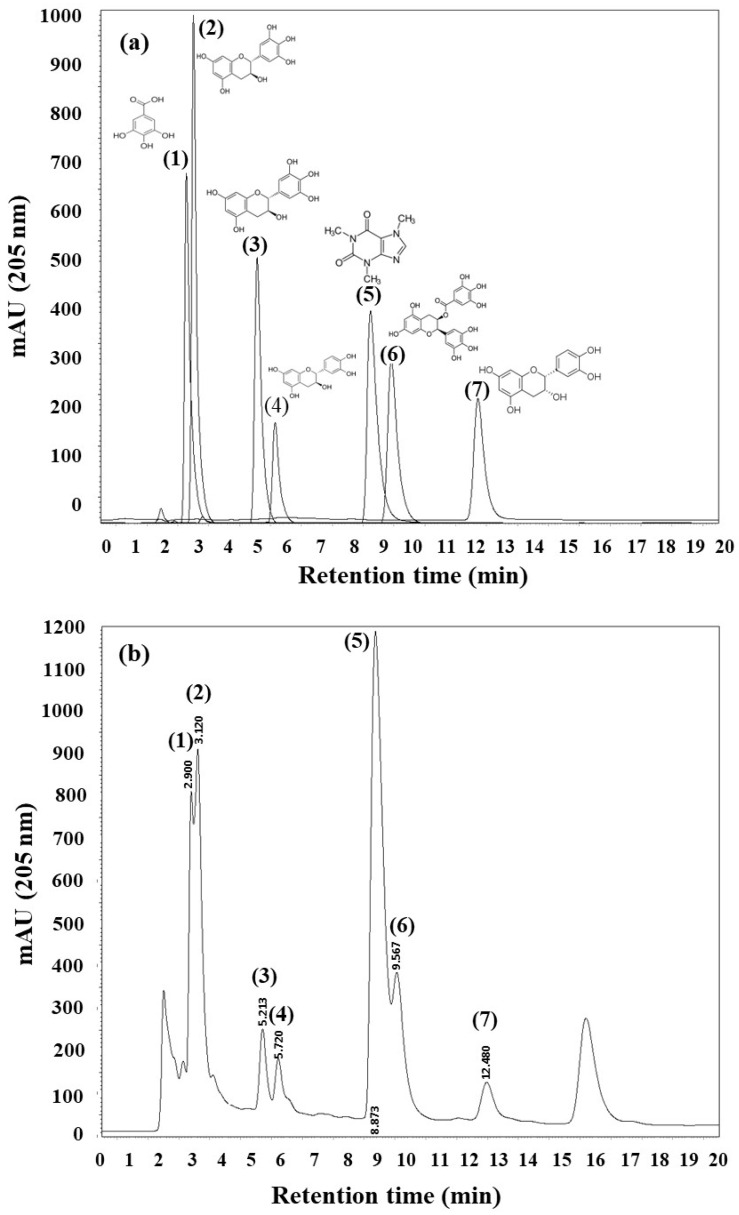

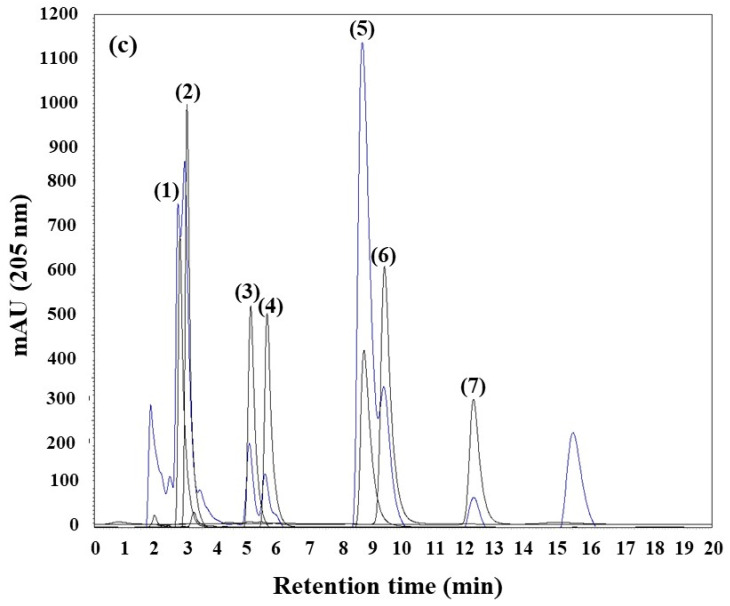
Chromatographic profiles of the results obtained on a C-18 column coupled to HPLC at 205 nm for the aqueous extract of RGT (**a**), the standards (**b**), and an overlay chromatographic profile (**c**). The black line corresponds to the aqueous extract of RGT. The peaks in blue represent the seven standards associated with RGT with respect to retention time: (**1**) gallic acid, (**2**) (−)-gallocatechin, (**3**) (−)-epigallocatechin, (**4**) (−)-catechin, (**5**) caffeine, (**6**) (−)-epigallocatechin gallate and (**7**) (−)-epicatechin.

**Figure 2:**
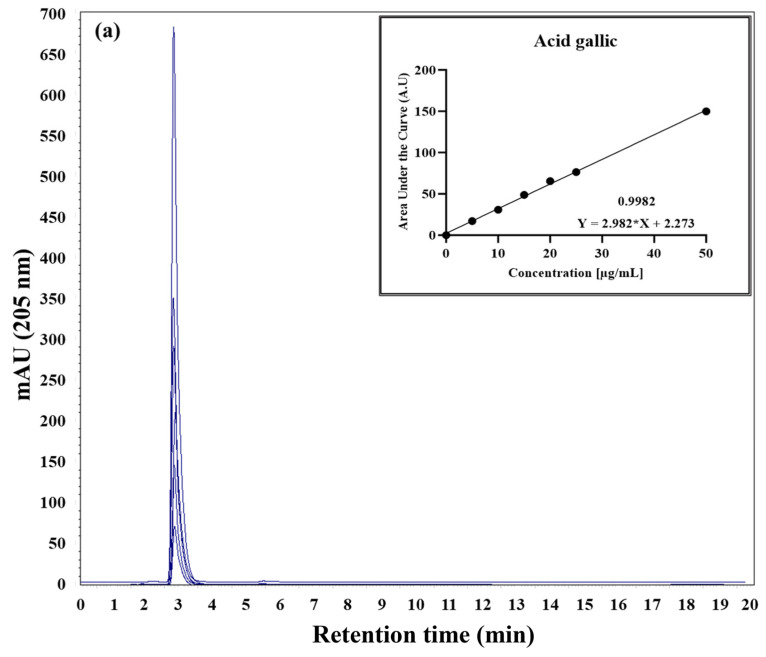

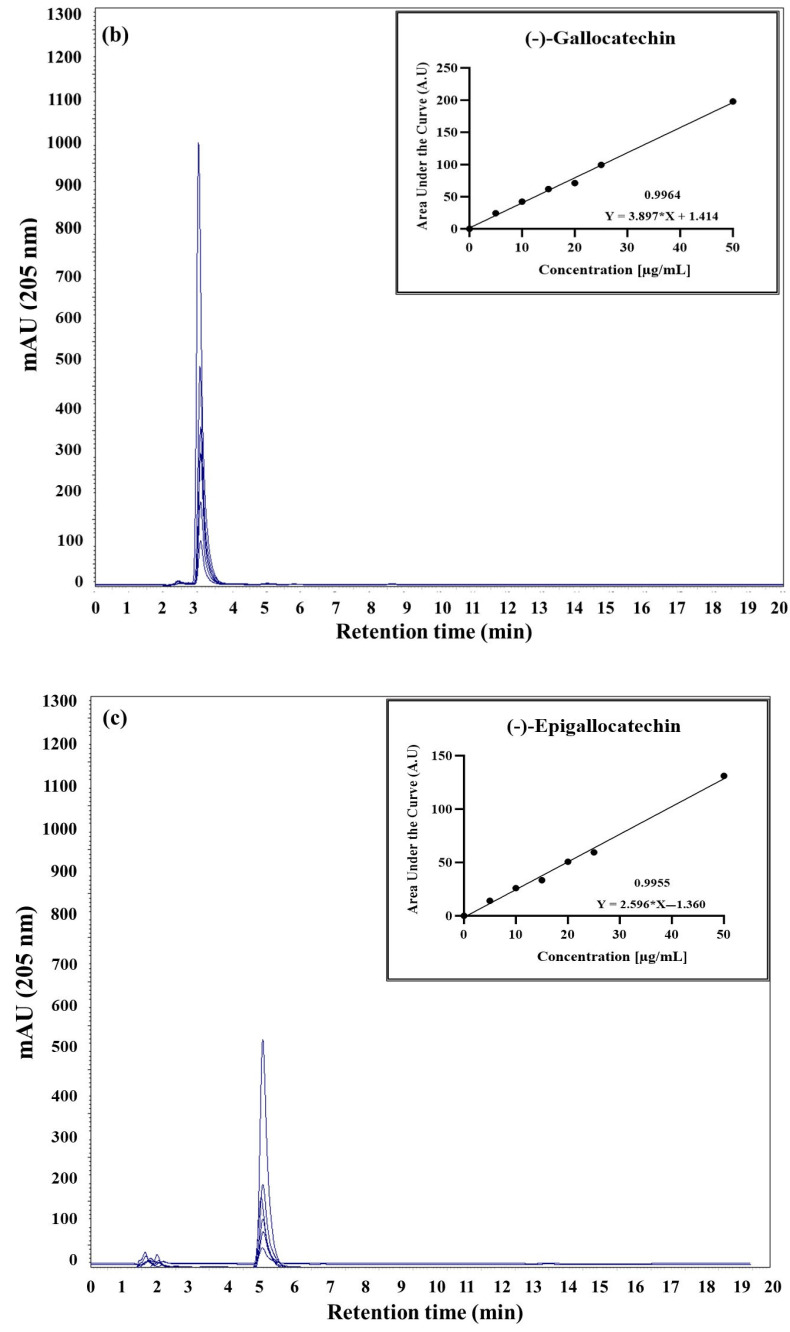

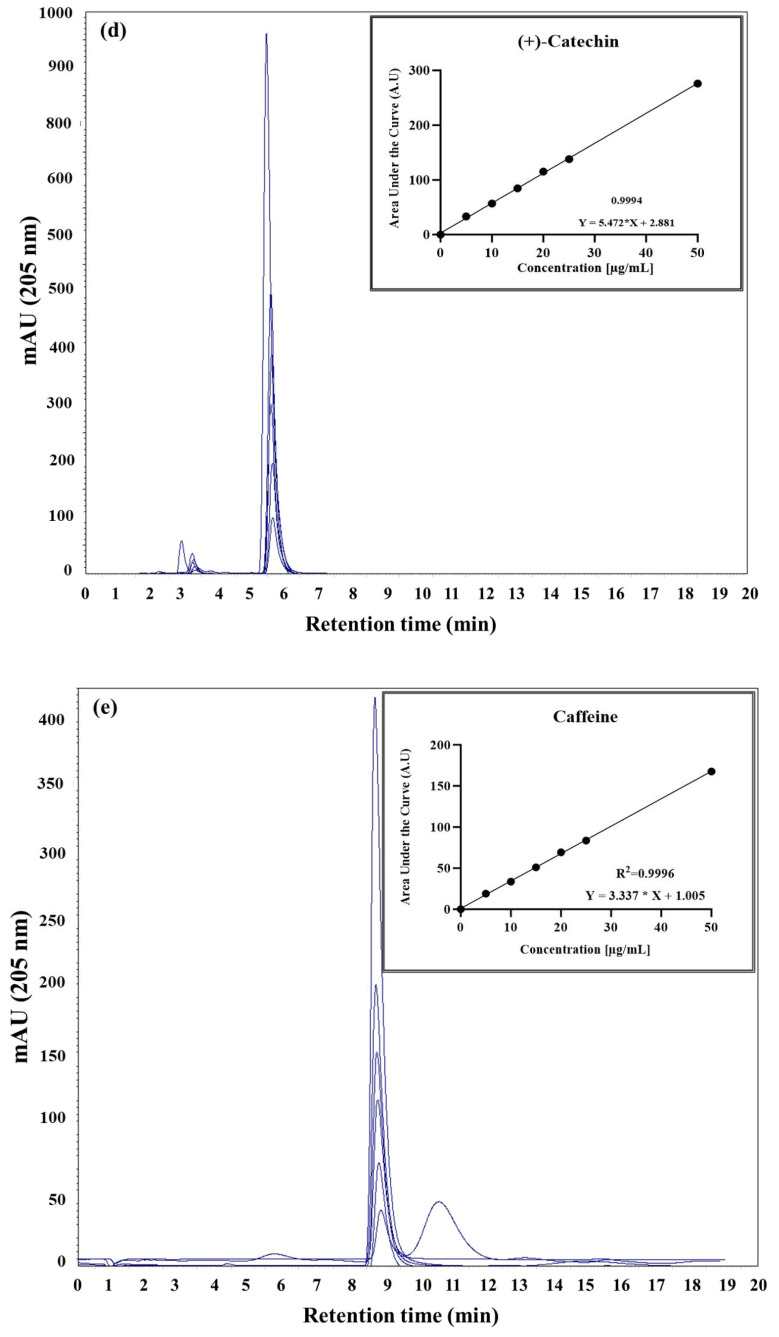

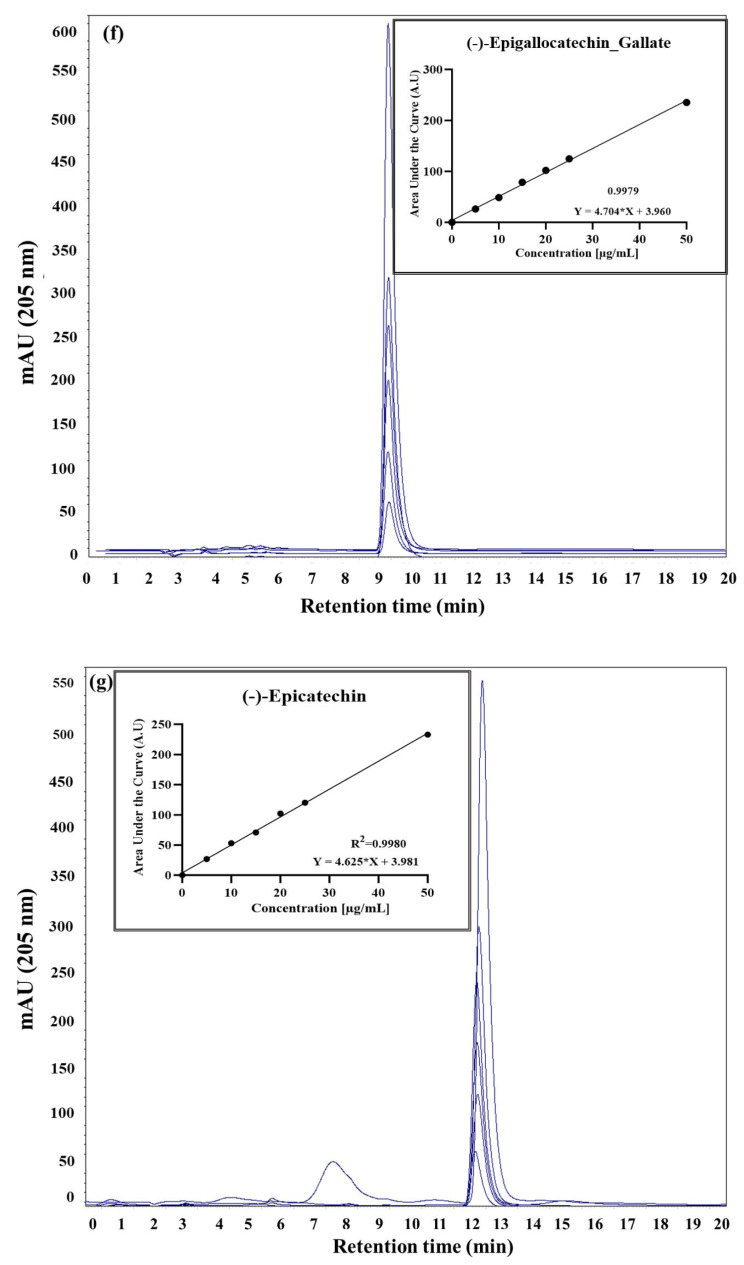
Chromatographic profiles for obtaining area under the curve values for six different concentrations of each of the seven identified metabolite in RGT. The obtained area under the curve values (mAU at 205 nm) were utilized to construct linear regression curves (inserts) enabling the precise quantification of the quantity of each compound present in one gram of RGT. The *y*-axis represents mAU obtained at 205 nm, while the *x*-axis corresponds to the retention time in min. (**a**) Gallic acid, (**b**) (−)-gallocatechin, (**c**) (−)-epigallocatechin, (**d**) (−)-catechin, (**e**) caffeine, (**f**) (−)-epigallocatechin gallate, and (**g**) (−)-epicatechin.

The superimposed chromatographic profiles for each of the standards and their retention time with respect to the aqueous extract of RGT area shown below (Figure 1). The seven chromatographic profiles (Figure 2) correspond to the seven standards of the identified components, each of which was injected at five different concentrations (50, 25, 20, 15, 10, and 5 µg/mL). The calibration curve represents the average of experiments conducted in triplicate. The area under the curve values was averaged and subjected to a statistical linear regression test with least squares adjustment, allowing for the determination of the related straight line (Figure 2, insert) and its function for each of the standards.

From these values, the amount of each of the compounds present in one gram of RGT could be determined (Table 1).

The values represent the concentration (mg/mL) of each identified compound in 1 g of RGT. The compounds are arranged in order of elution. Caffeine represents the component with the highest amount (312.77 mg/L), and epicatechin (29.82 mg/mL) represents the lowest concentration.

High-performance liquid chromatography has been used for many years as an analytical method that is easy and accessible for determining the presence of catechins in non-RGT. In our study, we employed an isocratic elution system with modifications, based on the research conducted by Wang et al. (2000) [19], using a simple mobile phase containing methanol, water, and orthophosphoric acid. This method allows for the separation of gallic acid and caffeine, which, while not belonging to the catechin group, hold biological significance. The chromatographic profiles presented in this study provide a detailed view of the results obtained using a C-18 column coupled to HPLC with a wavelength of 205 nm. These profiles allow for the identification of the components present in the aqueous extract part of RGT (Figure 1a), the standards (Figure 1b), and an overlaid chromatographic profile (Figure 1c). It is important to note that, although the chromatographic profile corresponding to the aqueous extract of RGT shows eight peaks, our focus was solely on the identification of biologically relevant components with high antioxidant potential [20,21,22,23,24]. The results obtained regarding the profile and elution times of these components are consistent with previous research [19]. Regardless of the separation method employed, the components eluted in the same order (gallic acid, (−)-gallocatechin, (−)-epigallocatechin, (−)-catechin, caffeine, (−)-epigallocatechin gallate, and (−)-epicatechin). These chromatographic profiles also provide crucial information about the separation and retention of these compounds in the column, which was essential for conducting both the qualitative and quantitative analyses performed after their identification.

The quality of RGT, is gaining significant importance in the food industry. It is essential to achieve that characteristic roasted aroma while preserving the widely recognized beneficial properties of GT [25]. When comparing Matcha GT with hōjicha (RGT), significant differences in their chemical composition become evident. RGT, tends to exhibit lower levels of catechins and lacks the presence of theanine and L-theanine, which are enantiomers found in conventional GT. In contrast, hōjicha displays relatively higher levels of (−)-Catechin and (−)-Gallocatechin gallate, along with the presence of (−)-Epicatechin and (−)-Epigallocatechin gallate, compounds that undergo epimerization [26]. These findings are based on the identification of compounds using reference standards.

One of the characteristics of RGT is a decrease in caffeine content due to sublimation reactions, making it more tolerable for individuals sensitive to this metabolite. Our findings regarding the elution pattern of compounds in RGT are compared to those obtained by Morikawa et al. (2019) [27], in which it is observed that the epimerization of catechins results in the generation of oligomeric products, which were detected with a baseline amplification.

The results obtained through HPLC demonstrated efficient separation and identification of the components, facilitating the precise quantification of each of them. The construction of standard curves played an essential role in allowing us to assess the linearity of the method, indicating that the detector’s response was proportional to the standard’s concentration within a specific range, as illustrated in Figure 2. The quantification of components using the HPLC method is widely recognized and employed in various research areas. For example, Zhang et al. (2015) [28] utilized this method to quantify and characterize sugars in 29 varieties of dates harvested in Saudi Arabia. Likewise, it has been applied in the quantification of sugars and organic acids in tomato fruits [29], and the quantification of fructooligosaccharides [30], but it has not been applied to RGT until now.

### 2.2. UHPLC-ESI-QTOF-MS/MS Analysis

**Figure 3:**
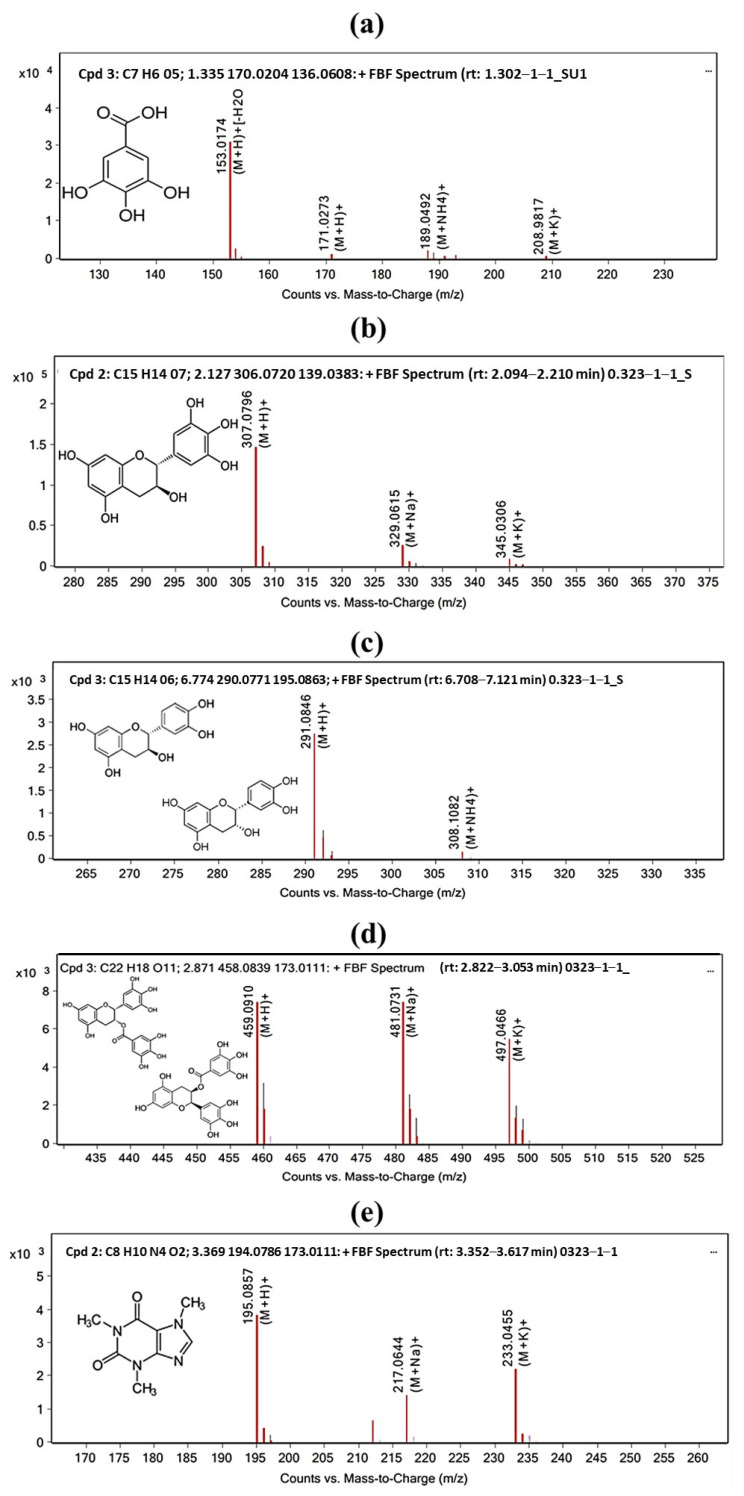
Mass spectra of detected ions for each identified compound. Five spectra are shown, each representing one of the identified compounds. The spectra illustrate ion intensity on the *y*-axis and mass-to-charge ratio (*m*/*z*) on the *x*-axis. The isotopic ion, which for all components was (M+H)+, indicates that the analyzed ion had a positive charge formed from the molecule (M) by adding a proton (H+). Abundance, the proportion of ions of different masses in a sample; in this case, the abundance of the peak corresponding to the associated compound’s *m*/*z* is represented. (**a**) Gallic acid, (**b**) gallocatechin, (**c**) catechin and epicatechin, (**d**) epigallocatechin and epigallocatechin gallate, and (**e**) caffeine.

The results obtained from mass spectrometry allowed us to clearly identify five of the individual components previously identified by HPLC through the *m*/*z* ions. In Table 2 and Figure 3, the five mass spectra obtained after analysis are depicted. These spectra graphically describe the ions detected by the mass spectrometer, with ion intensity on the *y*-axis and mass-to-charge ratio (*m*/*z*) on the *x*-axis. In each spectrum, the peaks of masses are observed in red, corresponding to the signals of individual ions detected in the mass spectrum. Each mass peak represents an ion with a specific mass-to-charge ratio. The total mass of the identified molecules is also indicated, which can be determined from the mass peaks in the spectrum. Additionally, the Z value is indicated, which corresponds to the ratio between the charge of an ion and its mass. Z is defined as the ion charge divided by its mass number, i.e., Z = q/m, where q is the ion charge, and m is its mass number. In other words, Z represents the number of electric charges that an ion carries per unit mass. For the identified components, the Z value was 1 in all cases, indicating that the ion in question carries a single elementary charge, often attributed to the presence of an ionized proton (H+). The fragmentation of this molecular cation into smaller ions can provide additional information about the molecule’s structure and identity.

Regarding the abundance value in mass spectrometry, it refers to the relative frequency or intensity of each ion detected in the mass spectrum. Abundance is expressed as a relative value indicating the proportion of ions with a specific mass in relation to other ions present in the sample. More abundant ions are detected as higher peaks, while less abundant ions are represented by lower peaks. This pattern is observed in the red peaks of each spectrum. Ion abundance provides information about the composition of the sample and the structure of the present compounds. The presence of intense peaks in the mass spectrum indicates the existence of important compounds in the aqueous extract of RGT.

The isotopic ion for all components was (M+H)+, signifying that the analyzed ion had a positive charge formed from the molecule (M) by adding a proton (H+). This is known as electrospray ionization. The (M+H)+ ion is one of the most common ions in mass spectrometry of organic compounds, as the addition of a proton efficiently ionizes neutral molecules and makes them detectable, with their mass and *m*/*z* ratio being unique to each compound.

The combination of an HPLC analysis followed by UHPLC-ESI-QTOF-MS/MS analysis stands as a highly relevant and scientifically valuable analytical strategy. Although the HPLC technique successfully achieved the separation of components present in RGT, it lacks the capability to provide detailed information regarding the identity of these compounds. The UHPLC, when coupled with mass spectrometry, delivered even more efficient separation and, most importantly, enabled the precise identification of the seven components present based on their masses and mass-to-charge ratios (*m*/*z*). This step is crucial in the current research as one of the objectives is the application of RGT nanotechnology. Accurately identifying these key components is crucial for understanding the synthesis and properties of AgNPs, such as those derived from RGT. This information forms the basis for optimizing the synthesis processes and improving the performance of NPs in various applications. It is essential for enhancing the effectiveness and stability of nanoparticle-based formulations, which can lead to the development of more efficient and innovative nanotechnology products.

In total, 5 spectra was obtained by UHPLC-ESI-QTOF-MS/MS. (+)-Catechin and epicatechin are molecules that share the same mass-to-charge ratio of 290.0771; they are considered enantiomers, meaning they share the same molecular formula, molecular mass, and charge but differ in spatial configuration and are non-superimposable mirror images of each other [31]. They possess identical physical and chemical properties except for their interaction with polarized light and their ability to interact with other chiral compounds. Enantiomers can be distinguished using chiral ionization techniques, generating ions with different mass-to-charge values for each enantiomer [32].

In this study, concerning RGT, the molecule catechin has been identified as (−)-Catechin, which is also a byproduct of (−)-Epicatechin generated through the oligomerization process [27]. As for the molecules corresponding to (−)-Epigallocatechin gallate and (−)-Epigallocatechin (Figure 3c), they have been identified as isomers with a mass-to-charge ratio of 458.0822. Being isomers, these molecules share the same molecular formula and molecular mass but differ in the arrangement of atoms within their molecular structure. They also possess distinct physical and chemical properties [33]. In mass spectrometry, isomers can be differentiated using collision-induced energy fragmentation (CID), where precursor ions collide with a collision gas and break into smaller ions [34]. The fragmentation pattern of a precursor ion depends on its molecular structure; thus, isomers can have different fragmentation patterns.

Finally, a second oligomerization product was identified, (−)-Gallocatechin (Figure 3b). Unlike catechin and epicatechin, this oligomerization product had a lower mass than the original monomer, (−)-Epigallocatechin (Figure 3d) [27]. This result is because oligomers can have molecular weights like or even greater than that of their original monomer, depending on how the oligomerization reaction was carried out and how many monomers were joined. However, regardless of the mass, the retention time will always be the same under the same conditions. 

### 2.3. Antioxidant Activity

To evaluate the ability of RGT to neutralize free radicals and its potential to prevent oxidative stress, a Trolox equivalent antioxidant capacity assay was performed. The antioxidant capacity of the RGT extract was compared to a Trolox standard curve, with results expressed in Trolox equivalents (ranging from 4 to 20 nmol/well). The analysis concluded that 0.1 g of roasted green tea exhibited an antioxidant capacity equivalent to 4 nmol of Trolox.

The demonstrated capacity of RGT to neutralize free radicals suggests a potential for protecting cells and tissues from damage caused by oxidative stress. This may have significant implications for overall health and specifically for oral health, as oxidative stress has been associated with various oral conditions [35,36,37,38,39].

Trolox is a chemical compound used as a reference standard in assays and studies that assess the antioxidant capacity of various substances, including foods, plant extracts, chemical compounds, and other products [40]. Trolox is a synthetic form of tocopherol, which is one of the forms of vitamin E [41]. The primary reason for its use as a reference is its greater stability compared to some natural antioxidants, such as polyphenols found in foods and plant extracts [20]. This stability means that it does not oxidize as easily when exposed to oxygen and light, making it suitable for laboratory assays. Therefore, Trolox allows for the standardization of antioxidant assays, ensuring that results can be more reliably compared.

In the present study, it was stated that 0.1 g of RGT is equivalent to 4 nmols of Trolox in terms of antioxidant capacity. This provides a relative measure of the tea’s antioxidant capacity compared to the reference standard Trolox. The antioxidant capacity was evaluated through a redox reaction based on the transfer of electrons and hydrogen atoms. In this redox reaction, the antioxidants present in RGT (phenols and flavanols) transfer electrons to the metal ion present in the Folin-Ciocalteu’s reagent. This redox reaction occurs similarly during the synthesis of AgNPs, acting as reducing agents in the neutralization of ROS and the reduction of the metal ion. In plants, the greater the antioxidant capacity, the higher the quantity of nanoparticles synthesized [42]. To date, there are few studies that evaluate the antioxidant effect of a substance in isolation. Most studies are comparative, either between different tea brands [43,44] or different plants [45,46] substances such as carotenoids [47]. However, in the present study, the aim was to determine if this effect exists and at what level, independently of other tea brands or plant substances. 

### 2.4. Biosynthesis of AgNPs, Hydrogel and Characterization

**Figure 4:**
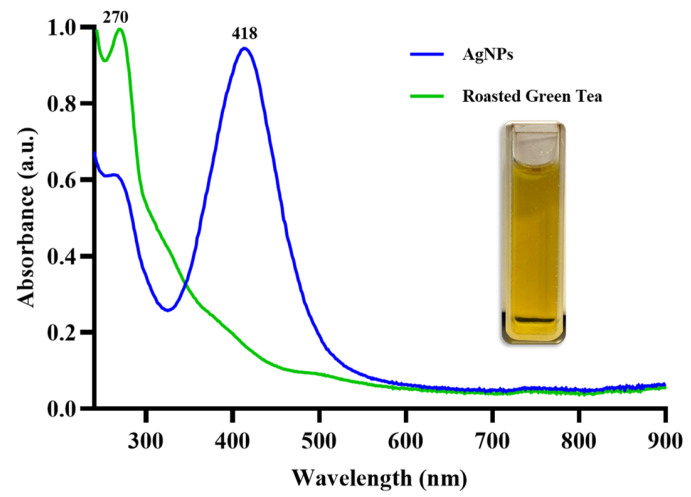
UV–Vis spectrum of synthesized silver nanoparticles. The surface plasmon resonance band in blue is observed at 418 nm, indicating the formation and stability of the AgNPs in the solution. This characteristic peak confirms the presence of well-dispersed silver nanoparticles and suggests a uniform particle size. The green band corresponds to the reducing agent, the aqueous extract of RGT, the surface plasmon resonance band in blue is observed at 270 nm.

**Figure 5:**
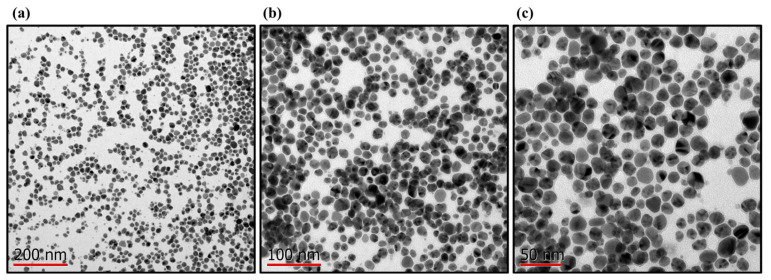
TEM micrographs of the synthesized silver nanoparticles, showing different levels of resolution. (**a**) Micrograph at 200 nm, providing an overview of the distribution and dispersion of the nanoparticles. (**b**) Micrograph at 100 nm, allowing for a more detailed observation of the morphology and size of the nanoparticles. (**c**) Micrograph at 50 nm, offering a high-resolution view of the crystalline structure and defined edges of the nanoparticle.

**Figure 6:**
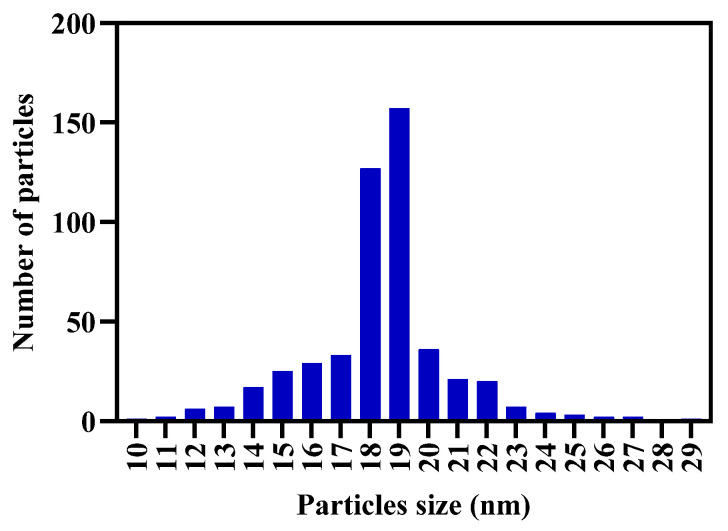
Histogram of the size distribution of the synthesized silver nanoparticles, using ImageJ software. The analysis was conducted on a total of 500 nanoparticles, taken from three micrographs (Figure 5a), showing a narrow size distribution with an average diameter of 19.85 ± 3 nm. The uniform size distribution indicates efficient and controlled synthesis of the nanoparticles.

The optimized conditions for the synthesis of AgNPs showed a color change in the aqueous extract of RGT, from brown to amber yellow. In the UV–Vis results, the surface plasmon resonance (SPR) of the AgNPs was observed in the range of 418 nm (Figure 4). The position of this absorption band provided an approximation of the size of the silver nanoparticles being obtained, also suggesting the formation of spherical or quasi-spherical. The synthesized were analyzed using TEM to determine their size, shape, and morphological distribution. TEM images revealed that the AgNPs predominantly exhibited a spherical morphology with a uniform size. Results were obtained for over 500 nanoparticles. The analysis of the nanoparticles was carried out using the ImageJ software (2023 version 1.54 h), and the histogram construction (Figure 5) was done using Prisma 10 for Windows 64-bit, version 10.3.1. The analysis determined that the nanoparticles have an average diameter of 19.85 ± 3 nm, with a narrow size distribution (Figure 6). The nanoparticles were well dispersed with no significant evidence of aggregation, suggesting good colloidal stability.

**Figure 7:**
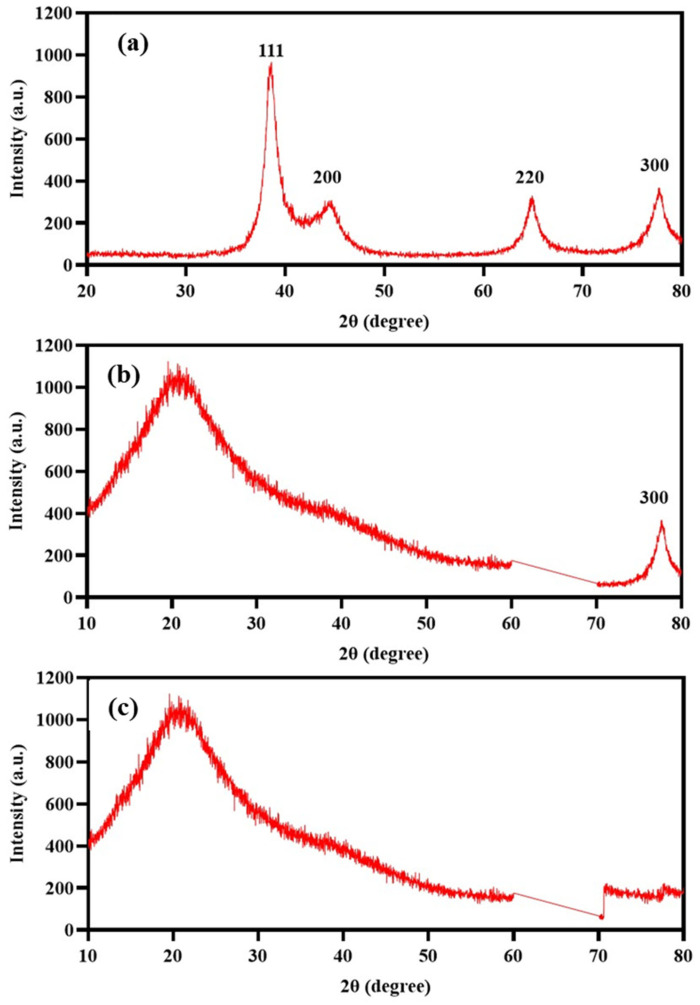
X-ray diffractograms (XRD) of the Gel-Alg-AgNPs hydrogel. (**a**) Silver nanoparticles (AgNPs): The peaks observed at 2θ values of 38.1°, 44.3°, 64.4°, and 77.5° correspond to the (111), (200), (220), and (311) crystalline planes of the face-centered cubic (FCC) structure of silver. (**b**) Hydrogel with AgNPs (Gel-Alg-AgNPs): The diffractogram shows a broad peak around 2θ ≈ 20°, indicating the amorphous nature of the hydrogel matrix. Additionally, a sharp peak at 2θ ≈ 78° confirms the presence of crystalline nanoparticles within the hydrogel. (**c**) Hydrogel without AgNPs (Gel-Alg): Only the broad peak at 2θ ≈ 20° is observed, suggesting the material is amorphous without the incorporation of silver nanoparticles.

The X-ray diffractogram presented in Figure 7 shows the diffraction patterns of the AgNPs. The peak at 38.1° (111 plane) is the most intense peak in the diffractogram, indicating that the 111 plane is the most predominant. The peaks at 44.3° and 77.5° (200 and 311 planes) correspond to the face-centered cubic (FCC) structure of the AgNPs. Good crystallinity is indicated by the presence of the peak at 64.4° (220 plane). The diffraction pattern found corresponds to the crystallographic card 040783.

On the other hand, Figure 7b shows the X-ray diffraction pattern of the Alg-Gel hydrogel modified with AgNPs. In this pattern, we can observe that the broad signal around 20–24°θ is closely related to the amorphous structure of the material. However, in the same diffractogram, a signal corresponding to the (300) plane family associated with the presence of crystalline material or AgNPs can be observed. It is important to note that the other signals associated with AgNPs observed in Figure 7b were not evident, probably due to an overlap of the amorphous signals of Alg-Gel.

**Figure 8:**
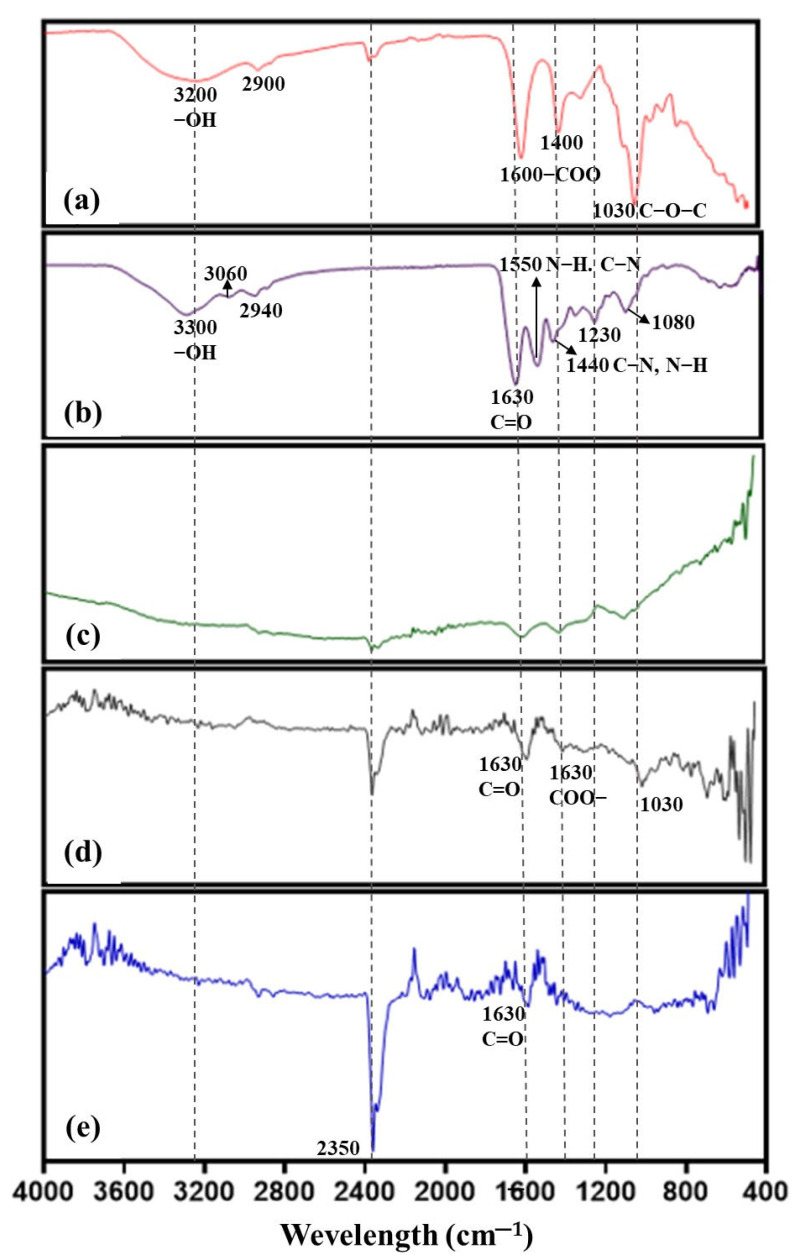
FTIR spectra of the different components of the Alg-Gel-AgNPs hydrogels: (**a**) alginate, (**b**) gelatin, (**c**) AgNPs, (**d**) Alg-Gel hydrogel, and (**e**) Alg-Gel-AgNPs hydrogel. The characteristic bands are observed in the different regions. The dashed lines indicate the positions of the bands. The *x*-axis represents the wavelength.

**Figure 9:**
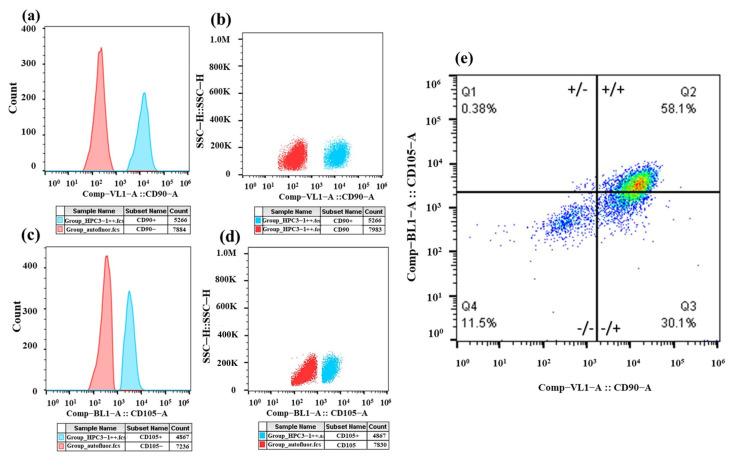
Flow cytometric analysis of SHEDs. Cells at 90% confluence (4 PDL) were cultured for 48 h. (**a**,**b**) The population labeled with anti-CD90 antibody showed positivity, as did the population labeled with anti-CD105 antibody. (**c**,**d**) The red/orange color represents the negative cell population (autofluorescence), while the blue color indicates the positively labeled cells. (**e**) The double-positive population for CD105 and CD90 accounted for 58.1%. Antibodies used: anti-human CD90 Super Bright 436 and anti-human CD105 Alexa Fluor. SHEDs = stem cells from human exfoliated deciduous teeth; PDL = population doubling level.

FTIR analysis was performed to determine the chemical compositions, and functional groups present in the hydrogel, providing essential information about the chemical nature of each component. Figure 8 shows the obtained spectra: (a) alginate, (b) gelatin, (c) AgNPs, (d) hydrogel without AgNPs, and (e) hydrogel with AgNPs. Based on the literature, an analysis of the functional groups present in each component was conducted. The peak at 3200 cm^−1^ in the alginate spectrum is attributed to O–H stretching vibrations, while the peak at 2900 cm^−1^ indicates C–H stretching. In the same spectrum, peaks at 1600 cm^−1^ and 1400 cm^−1^ correspond to C=O (COO–) stretching and C–H bending vibrations, respectively. The peak at 1030 cm^−1^ is related to C–O–C stretching vibrations. In the case of gelatin (Figure 8b), peaks at 3060 cm^−1^ and 2940 cm^−1^ correspond to N–H and C–H stretching. The peak at 1630 cm^−1^ is attributed to C=O stretching, and peaks at 1550 cm^−1^ and 1440 cm^−1^ correspond to N–H bending and C–N stretching, respectively. Finally, peaks at 1230 cm^−1^ and 1080 cm^−1^ are attributed to C–N and N–H bending vibrations. In the spectrum of the AgNPs synthesized with RGT (Figure 9c), a peak around 1630 cm^−1^ is observed, indicating C=O stretching, attributed to the presence of organic compounds from RGT that have adhered to the surface of the AgNPs. The lack of other prominent peaks suggests a simplified structure compared to the polymers. The hydrogel without AgNPs (Figure 8d) shows characteristic peaks around 1630 cm^−1^ and 1530 cm^−1^, corresponding to C=O and COO– stretching, respectively. The peak at 1030 cm^−1^ is related to C–O–C stretching. Finally, the hydrogel with AgNPs (Figure 9e) displays a distinctive peak at 2350 cm^−1^, and the peaks at 1630 cm^−1^ indicate C=O stretching, consistent with the presence of both the hydrogel matrix and the incorporated AgNPs [48,49,50]. The biosynthesis of AgNPs using an aqueous extract of RGT was carried out through the reduction of silver ions by bioactive compound, which functioned as reducing agents. HPLC-MS results revealed that the aqueous part of the RGT contains phenols, such as (−)-Gallocatechin, (−)-Epigallocatechin, (−)-Catechin, (−)-Epigallocatechin gallate, (−)-Epicatechin, gallic acid, and caffeine which act as reducing and coverage agents in the synthesis of AgNPs. This has been widely reported in many studies [51,52,53,54]. The presence of these components is not only important, but so is the quantity available, as the shape and size of the nanoparticles are documented to be dependent on the characteristics of the reducing agent [16]. Data indicated that at a higher concentration of RGT (10-4%), the UV-Vis spectrum showed a very broad plasmon surface, suggesting that the AgNPs were larger or perhaps agglomerated. This mechanism is common, as concentration affects the reduction kinetics and the stabilizing properties of the resulting AgNPs [55]. 

Therefore, it is important to highlight that the 2% aqueous extract of RGT (quantified by HPLC, Figure 2-Table 2) in our research allows us to achieve a more controlled chemical composition on the surface of the AgNPs, which influences their interaction with biological systems. This results in a stable synthesis with AgNPs sizes ideal for biomedical applications [56]. During the reaction, a color change from brown to amber yellow was observed, as demonstrated in the quartz cuvette inserted in the UV-Vis spectrum (Figure 4). This change occurs due to the SPR of AgNPs. When Ag^+^ in the solution is reduced to Ag^0^ and the nucleation process forms NPs, the free electrons on the surface of the nanoparticles oscillate collectively in response to incident light [57]. This resonant oscillation absorbs certain wavelengths of light and scatters others, resulting in the color change [58]. While AgNPs formation can exhibit a variety of colors, amber yellow is associated with smaller and spherical NPs [59]. 

In comparison, syntheses that tend to produce a gray or even green color are usually related to larger NPs [15,60]. UV-VIS analysis was used to confirm the formation of AgNPs, and our results showed an absorption peak around 418 nm, indicating the formation of AgNPs. This result is consistent with various studies using GT (not roasted) as a reducing agent and obtaining a plasmon surface between 410 and 450 nm, with characteristics like ours [18,61,62]. Compared to other studies, the use of RGT provided a stable synthesis and based on transmission micrographs, yielded a large quantity of dispersed, quasispheroidal NPs. In Figure 5, three micrographs with different pixel distances clearly show the size and shape of the AgNPs. It is observing a correlation with the UV-Vis result (418 nm) as we are visually obtaining nanometric structures with uniform distribution, which is essential for optical, electronic, and catalytic properties. Uniformity in AgNPs synthesis minimizes aggregation, which can negatively impact the function of AgNPs. In XRD analysis, diffraction patterns showed distinct peaks at 2θ positions of 38.1°, 44.3°, 64.5°, and 77.5°, corresponding to the (111), (200), (220), and (311) crystallographic planes of metallic silver with a face-centered cubic (FCC) structure. These results align with standard data (JCPDS no. 04-0783) [63], confirming the crystalline nature. The most intense peak at 38.1° (111) is the preferred growth plane of NPs, a common trait in AgNPs, as it directly impacts lower surface energy on this plane [64]. 

The NP size was also confirmed by XRD results using the Scherrer equation, which estimates that the AgNPs are around 15-20 nm, reinforcing the strong correlation between good crystallization of the silver and uniformity in distribution and size [65,66]. The incorporation of AgNPs into the Alg-Gel hydrogel, based on XRD results, was evident through a broad band associated with the presence of an amorphous material. This finding is consistent with various studies where it is not possible to clearly see the peaks of metallic or silver nanoparticles due to the matrices overlapping the diffraction patterns. However, the peak at plane 311 can be observed, confirming the presence and successful incorporation of AgNPs into the hydrogel matrix without a reduction in this peak, suggesting that this incorporation does not negatively affect the crystallinity of the AgNPs. Maintaining the integrity of the AgNPs once mixed with alginate and gelatin is crucial for maintaining their biological application, where, in addition to the effect, good structural stability is required [67]. FTIR analysis helped identify the functional groups present on the surface of AgNPs, the components of the Alg-Gel hydrogel, and the matrix with AgNPs. It is important to note that the correlation with the peaks of alginate and gelatin was consistent with previous reports [68]. In this type of material, the peak at 3200 cm^−1^ corresponding to −OH stretching and at 2900 cm^−1^ to CH stretching are frequently observed, associated with compounds rich in hydrogen bonds and hydroxyl groups. Together with the other peaks, they confirm the chemical structure of alginate [68]. Regarding gelatin, amine groups are prominent and contribute to biological functionality and compatibility [69]. 

In the FTIR corresponding to AgNPs, no prominent peaks are observed, which can be explained by the inorganic nature of AgNPs [70]. Unlike organic compounds, which have many covalent bonds, AgNPs have metallic bonds that do not absorb in the infrared spectrum in the same way [71]. Some regions of the spectrum may reveal very small peaks that would be associated with a very thin layer of organic compounds derived from RGT, and any signal that might appear could be overlapped with the signals of the hydrogel matrix, as observed in the spectra in Figure 8. The peak at 1030 cm^−1^ is related to C–O–C stretching, indicating the presence of functional groups of alginate and gelatin already in the matrix [19,72]. These vibrations are characteristic of ester and carboxylate bonds, which are essential for forming the hydrogel. The appearance of the peak at 2350 cm^−1^ may be related to the interaction between the hydrogel matrix and the AgNPs, suggesting a possible chemical-physical interaction between these components. However, this peak could also be attributed to carbon dioxide absorption, indicating possible interactions with the environment during the synthesis process, as it is lower in the hydrogel without nanoparticles [73]. The results obtained have provided important data that allow us to ensure the quality and functionality of the AgNPs and their association with gelatin and alginate.

### 2.5. SHED Cells Characterization

The characterization of the primary SHEDs culture revealed positivity for CD90 and CD105 markers via flow cytometry analysis (Figure 9). The analysis displayed autofluorescence in red/orange, representing the negative cell population, and in blue, the positively labeled cells (Figure 9a–e). The results showed that 58.1% of the cells were double-positive for CD105 (Super Bright 436) and CD90 (Alexa Fluor), confirming the mesenchymal origin of the primary SHEDs culture.

### 2.6. SHEDs–AgNPs Dose-Response

**Figure 10:**
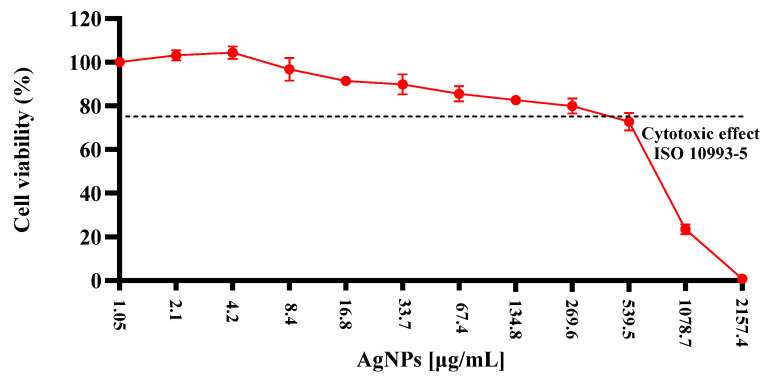
Cell viability of SHEDs treated with various concentrations of silver nanoparticles. The highest concentration tested was ~2157.4 µg/mL, with 12 successive 1:1 dilution to obtain a series of decreasing concentrations. The *x*-axis shows the different concentrations of AgNPs, from ~2157.4 µg/mL to ~1.05 µg/mL, while the *y*-axis indicates the percentage of cell viability. The dashed line represents the threshold for cytotoxic effect based on ISO 10993-5. The CC_75_ is observed around 320.6 µg/mL, where cell viability is reduced to 75%.

To determine the therapeutic dose of silver nanoparticles to be used in hydrogels, a cell viability assay was conducted to observe the effect of various concentrations of silver nanoparticles on SHEDs (Figure 10) corresponds to the results obtained, where starting from the highest concentration of 20 mM (equivalent to ~2157.4 µg/mL of AgNPs), 12 successive 1:1 dilution was performed to obtain a series of decreasing concentrations. The *x*-axis shows the different concentrations of AgNPs, from ~2157.4 µg/mL to ~1.05 µg/mL, while the *y*-axis indicates the percentage of cell viability, with 100% indicating complete viability without toxic effect, and decreasing values reflecting increased toxicity. The graph demonstrates a significant decrease in cell viability at higher concentrations of AgNPs, indicating high toxicity. As the AgNP concentrations are reduced through successive dilutions, cell viability gradually increases, suggesting a lower level of toxicity at these lower concentrations.

The use of AgNPs in biomedicine has been widely reported in various health fields [74]. However, it is important to consider that their use can have not only positive effects; AgNPs may also have negative impacts due to their toxic effects. The cytotoxicity of AgNPs can be categorized into two forms: endogenous, which considers factors such as size, shape, and even functional groups adsorbed on the surface, and exogenous factors, such as the dosage used [75]. In our study, the cytotoxic effect of AgNPs on SHEDs was evaluated to determine the therapeutic dose suitable for subsequent combination with alginate and gelatin. The observed toxicity of AgNPs is primarily linked to the release of silver ions into the cells, which can induce oxidative stress and damage cellular components, ultimately affecting cell viability. The determination of a safe and effective dose is crucial for leveraging the benefits of AgNPs in tissue engineering while minimizing potential cytotoxic effects [76]. 

In most cell types, the absorption of AgNPs occurs through endocytosis, with endosomes and lysosomes being the target organelles [77]. When AgNPs reach these organelles, the cellular microenvironment acidifies, leading to the production of reactive oxygen species (ROS), which causes oxidative damage at the protein and DNA levels, followed by mitochondrial dysfunction [78]. Protein damage also results in defective or discontinuous membranes, leading to cytoplasmic leakage and subsequent necrosis, while the rupture of lysosomal membranes activates lysosome-mediated apoptosis [79]. 

The size of AgNPs is closely associated with the degree of toxicity, as it determines reactivity [80]; AgNPs smaller than 10 nm are more toxic than those of larger size. This effect may be related to biological transport at the cellular level. Larger AgNPs can enter and exit cells through ion channels, while smaller AgNPs can cross the cell membrane and act directly on the cell [81]. Smaller AgNPs technically have a larger surface area in any biological system, giving them the ability to distribute more widely in the target organ, leading to significant damage [82]. This finding has been observed in several studies, such as that of Park et al. (2010), where mice were orally exposed to 1 mg/kg of AgNPs and nanoparticle dissemination was found in organs such as the brain, liver, and kidneys. The nanoparticles had sizes of 22 nm [83]. Using 10 nm AgNPs administered intraperitoneally, histopathological changes such as cellular necrosis and focal necrosis in the liver were observed after 6 h [84]. Although many efforts have been made to determine the toxic effect of AgNPs, it is important to highlight that there is a direct association with the synthesis method and the agents present in the solution, so each case should be evaluated independently. Our results indicated an inverse relationship between AgNPs (18 nm) concentration and cell viability, consistent with findings in other studies evaluating cytotoxic effects. As the concentration of AgNPs decreases through successive dilutions, cell viability increases due to the reduction in toxicity. This suggests the presence of a therapeutic window where AgNPs can be used effectively without compromising cell integrity, which is critical for their safe application in biomedical contexts.

### 2.7. Indirectly Cytotoxicity Evaluation of Alg-Gel Hydrogels Containing AgNPs

**Figure 11:**
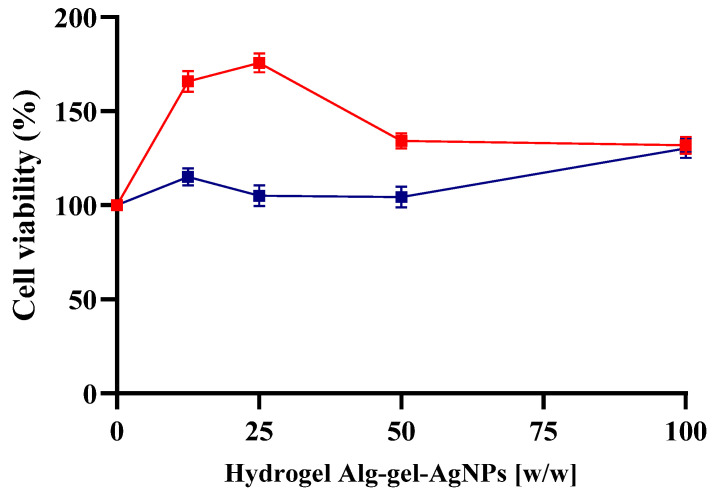
Cell viability of SHEDs treated with alginate–gelatin–AgNPs hydrogels at various concentrations (100%, 50%, 25%, 12.5% *w*/*w*). Cell viability is measured as a percentage, where 100% indicates complete viability with no toxic effect. The red line represents the hydrogels with AgNPs, and the blue line represents the hydrogels without AgNPs. The results indicate that hydrogels containing AgNPs maintain high cell viability even at elevated concentrations, suggesting a lack of cytotoxicity and good cellular tolerance to the nanoparticles.

To determine that the incorporation of AgNPs (at therapeutic doses) into the hydrogel was not cytotoxic, a cell viability assay was conducted. This assay was performed with the hydrogel at concentrations of 100%, 50%, 25%, and 12.5% *w*/*w* (Figure 11). This was crucial to ensure that the hydrogel with AgNPs does not cause adverse effects on cells. The results of the cell viability assay showed that the hydrogel loaded with AgNPs maintained high cell viability, indicating low cytotoxicity. As the concentration of the hydrogel decreased, cell viability remained constant or even slightly increased, suggesting that the cells could tolerate the AgNPs well under these conditions. These findings indicated that the hydrogel with AgNPs is biocompatible and safe for use in biomedical applications at the tested concentrations.

### 2.8. Evaluation of the Hydrogel in Bone Differentiation Processes

**Figure 12:**
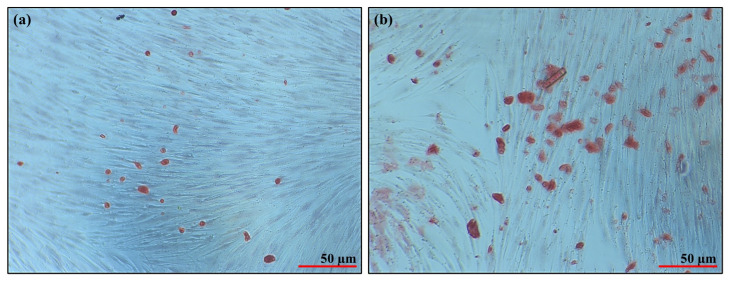
Alizarin Red staining of SHEDs cultured in hydrogels. (**a**) Hydrogel without AgNPs showing fewer calcium deposits, indicating lower osteogenic differentiation at 60×. (**b**) Hydrogel with AgNPs demonstrates increased calcium deposits, indicating enhanced osteogenic differentiation at 60×.

After obtaining positive results from the cell viability assay, the study progressed to the next phase, utilizing a hydrogel composed of 4% alginate (*w*/*v*), 2.5% gelatin (*w*/*v*), and 4 µg/mL of AgNPs (therapeutic dose). This phase aimed at evaluating the hydrogel’s ability to induce osteogenic differentiation in SHEDs. Osteogenic differentiation was assessed by detecting calcium deposits using Alizarin Red staining (Figure 12). The results revealed a significant increase in calcium deposition in SHEDs cultured within the hydrogel containing AgNPs compared to the control group without AgNPs. Alizarin Red staining confirmed that the hydrogel with AgNPs effectively promoted mineralization, highlighting its potential to enhance osteogenic differentiation.

The evaluation of Alg-Gel hydrogels containing AgNPs on SHEDs demonstrated a non-toxic effect. While the incorporation of silver nanoparticles into hydrogels has been extensively studied for its potential antimicrobial properties, ensuring their safety for human cells remains crucial. Integrating AgNPs into biomaterials enables localized and sustained release directly at the site of injury, positioning it as a highly promising pharmacological strategy for targeted therapeutic applications. [85]. Chen et al. (2021), L929 cells showed high viability and proliferation with ODex/HA-ADH and ODex/HA-ADH/HACC hydrogels (over 90%). In contrast, the Ag@ODex/HA-ADH/HACC hydrogel reduced cell viability (76.41%) and caused morphological changes, indicating cytotoxicity due to silver nanoparticles. However, it is important to highlight the need for assays to determine the therapeutic dose of AgNPs [86]. The study confirms that the Alg-Gel-AgNPs hydrogel can be safely used without causing harm to healthy cells. A primary goal of this hydrogel formulation is to induce the differentiation of SHED cells into an osteoblastic lineage, which is crucial for bone regeneration in areas with defects. This approach to bone regeneration emphasizes the biomaterial’s ability to promote progenitor cell differentiation (osteoinduction), support the proliferation of bone cells, and enhance the integration of the new bone with the surrounding tissues. This dual functionality—both promoting differentiation and aiding in integration—positions the hydrogel as a valuable tool in tissue engineering and regenerative medicine [87].

Hydrogels are highly valued in tissue engineering due to their biocompatibility and the ability to create a supportive environment for cell growth and differentiation. In this study, AgNPs were incorporated into an Alg-Gel matrix, which demonstrated the capability to promote the differentiation of stem cells from SHEDs into an osteogenic lineage. This was evidenced by the increase in calcium deposits, indicating successful mineralization as observed in the osteogenic differentiation assays (Figure 12b). The use of hydrogels in biomedical applications is particularly promising because they not only offer biocompatibility but also feature bioactive sites that can be functionalized for specific purposes, such as promoting tissue regeneration. Moreover, hydrogels can localize drug delivery, preventing the active compounds from entering the systemic circulation, which enhances therapeutic efficacy and reduces potential side effects. Various natural and synthetic polymers, including collagen, gelatin, sodium alginate, hyaluronic acid, and polyvinyl alcohol, have been used to fabricate hydrogels for different biomedical applications, further underscoring their versatility and importance in the field [88]. 

Hydrogels have been manufactured using one or more polymers, loaded with various elements such as drugs or nanoparticles. For example, the conjugation of chitosan/rosuvastatin nanoparticles in sodium alginate and polyvinyl alcohol for use as a controlled drug delivery system showed high cell viability in human fibroblasts [89]. Meanwhile, hyaluronic acid has been widely used in bone regeneration, particularly in dental and craniofacial fields [90]. Hybrid gels have been prepared, which often help to improve mechanical properties. Fang Guo et al. (2020), designed polyacrylic acid alginate (CPP/PAA-Alg) hydrogels and added calcium polyphosphate, obtaining good mechanical and osteointegration properties [91]. 

While it is important to have a biomaterial with osteoinductive capacity, AgNPs also regulate the antimicrobial load as their bactericidal effect against many bacteria has been widely demonstrated [56,92,93]. Recent research has increasingly emphasized the integration of tissue engineering and nanotechnology, particularly focusing on the role of silver nanoparticles (AgNPs) in promoting osteogenic differentiation. AgNPs release silver ions (Ag^+^), which can interact with stem cells and stimulate the expression of osteogenic genes, facilitating the differentiation process. The interaction of these silver ions with cellular components activates various signaling pathways that are crucial for osteogenesis. For instance, at a concentration of 4 μg/mL, AgNPs have been shown to promote osteogenic differentiation in urine-derived stem cells, a process that also involves the polymerization of actin and increased cytoskeletal tension through the activation of the RhoA signaling pathway. In our study, we observed similar effects, where AgNPs at the same concentration successfully promoted the differentiation of SHEDs into an osteogenic lineage. This finding underscores the potential of AgNPs as a therapeutic tool in tissue engineering, specifically in bone regeneration applications [94]. The evaluation of AgNPs on the osteogenic differentiation of human periodontal ligament fibroblasts (HPDLFs) demonstrated a dose-dependent enhancement of this process. Notably, the expression of RhoA, a key regulator of cytoskeletal dynamics and cell differentiation, was significantly upregulated, suggesting its involvement in promoting osteogenesis. However, when TAZ, a crucial transcriptional coactivator in the Hippo signaling pathway that influences osteogenesis, was silenced, the osteogenic differentiation was markedly attenuated, as evidenced by a reduction in alkaline phosphatase (ALP) activity. These findings underscore the intricate regulatory mechanisms at play, where AgNPs modulate osteogenic differentiation through the interplay of RhoA and TAZ pathways. The addition of these properties to existing biomaterials is advancing [95]. 

## 3. Conclusions

Nanotechnology has experienced significant growth due to its numerous applications, which has driven the development of various synthetic routes. However, many of these routes use toxic reagents, such as chemical synthesis, and require large amounts of energy (physical synthesis). With increasing concerns about environmental and health safety, an alternative is to employ more ecological methodologies, such as biological synthesis, which utilizes bacteria, fungi, plants, and other organisms. In this study, AgNPs were successfully synthesized using an RGT extract. The optimized conditions allowed the production of AgNPs with an average diameter of 19.85 ± 3 nm, demonstrating good colloidal stability. The integration of AgNPs into an alginate–gelatin hydrogel matrix and subsequent characterization revealed the presence of crystalline AgNPs and confirmed the interactions between the hydrogel components. The AgNP-loaded hydrogel, evaluated in terms of biocompatibility, demonstrated high cell viability, indicating that the hydrogel is safe for biomedical applications. Moreover, it promoted osteogenic differentiation in SHEDs, underscoring the significance of this study in advancing the integration of tissue engineering and nanotechnology. The combination of these fields could help medical practice and the biomedical industry, providing more effective and personalized solutions for tissue regeneration and the treatment of various pathologies. This study paves the way for future research focused on optimizing and expanding the use of biosynthesized nanoparticles in different biomedical applications. The combination of nanotechnology and tissue engineering could help develop new biomaterials with customizable properties that meet specific clinical needs, such as bone regeneration, soft tissue repair, and controlled drug delivery. Additionally, the use of natural extracts like roasted green tea is safe for use in green synthesis of nanomaterials, promoting sustainable practices in the biomedical industry. Future research should focus on determining the optimal concentrations of AgNPs in hydrogels to maximize osteogenic efficacy while minimizing potential toxicity, with evaluations in animal models to confirm the biocompatibility and efficacy of AgNPs-loaded hydrogels in a more complex biological environment.

## 4. Materials and Methods

### 4.1. Materials

Hōjicha Green Tea—Japanese roasted green tea (cultivated in Shizuoka, near Mount Fuji, 1.5 Oz)—at a concentration of 2% or 10% *w*/*v*; reverse-phase C-18 column coupled to HPLC with 4.6 mm internal diameter × 150 mm length HPLC system with a particle size of 3.5 µm (Agilent Technology, Zorbax, Santa Clara, CA, USA), 0.22 µm pore diameter PVDF membrane (Durapore^®^, Millipore, Merck, Darmstadt, Germany), methanol (CH_3_OH, HPLC-grade methyl alcohol, Meyer, CAS 67-56-1), deionized water (Milli-Q^®^ deionized H_2_O), orthophosphoric acid (H_2_PO_4_, ACS reagent Jalmek^®^ Scientific, San Nicolas Garza, Nuevo Leon, Mexico). Reference standards: (−)-gallocatechin, (−)-epigallocatechin, (+)-catechin, (−)-epigallocatechin gallate, (−)-epicatechin (Sigma-Aldrich, analytical standard of USA ≥90% HPLC), caffeine (Caffeine standard, Agilent Technology ≥90% HPLC, Santa Clara, CA, USA), gallic acid (TraceCERT^®^ Sigma-Aldrich Co. LLC, made in Switzerland CAS-No 149-91 7). A 100 µL chromatography syringe with a fixed needle (Hamilton 700 HPLC). UHPLC 1290 Infinity II system (Agilent Technologies) coupled to a QTOF 6545 mass spectrometer (Agilent Technologies), Zorbax Eclipse Plus C-18 column (2.1 × 50 mm; 1.8 μm, Agilent, USA). 20% *w*/*v* Na_2_CO_3_ (Sodium Carbonate) solution, Folin–Ciocalteu’s 2N phenol reagent (Folin and Ciocalteu’s phenol reagent 2 M Sigma-Aldrich, Germany), 5% *w*/*v* NaNO_2_ (Sodium Nitrite) solution, 10% *w*/*v* AlCl^3^ (Aluminum Chloride) solution, 1 M NaOH_2_ (Sodium Hydroxide) solution. Sodium alginate (Sodium alginate, natrium alginate algin, Sigma Aldrich, St. Louis, MO, USA), 50 mM calcium chloride, Total Antioxidant Capacity Assay Kit (Sigma-Aldrich, MAK187, St. Louis, MO 63103, USA), DMSO (Dimethyl sulfoxide, Karal, Leon, Gto, Mexico), 110 mm diameter Whatman membranes, Buchner funnel, graduated glass cylinder, electric hotplate, magnetic stirrers, thermometer. Agilent software packages: MassHunter Qualitative Analysis (Agilent Mass Hunter Qualitative Analysis (Qual) B.07.00), Agilent Profinder (Agilent Mass Hunter Profinder B.06.00), Agilent Mass Profiler Professional (Agilent Mass Profiler Professional (MPP) B.13.0). Sodium alginate (Sodium alginate, natrium alginate, Sigma Aldrich, St. Louis, MO, USA), MTT (Thiazolyl Blue Tetrazolium Bromide, Sigma-Aldrich) and microplate spectrophotometer reader (Multiskan Go, Thermo Fisher Scientific™, Helsinki, Finlandia).

### 4.2. Methods

#### 4.2.1. Obtaining the Aqueous Extract

Aqueous extract was obtained by combining 2 g of RGT and 100 mL of H_2_O (or 10 g for microdilution assay) at a boiling state (95 °C) and kept at boiling temperature for 10 min (with stirring). After 10 min, the infusion was allowed to cool to 25 °C. Filtering was carried out using two 110 mm diameter Whatman membranes placed in a Buchner type funnel. Subsequently, the aqueous extract of RGT underwent centrifugation (10,000 rpm, 20 °C, 20 min, using a benchtop centrifuge equipped with a fixed-angle rotor designed for 15 mL Falcon tubes). The supernatant pass through a second filtration process, through a vacuum filtration system operating at 220 V/50 Hz, was used in conjunction with a glass filter holder assembly using a PVDF membrane with a pore diameter of 0.22 µm. The aqueous extract of RGT obtained was stored at −20 °C until use for analysis.

#### 4.2.2. Identification of Metabolites and Quantification by HPLC

For the separation of the components found in the aqueous extract of RGT, a reversed-phase C-18 column coupled to HPLC was used. Initially, the equipment was washed and purged, and the column was equilibrated with the elution solution (CH_3_OH/H_2_O/H_2_PO_4_) in percentages of 20/79.9/0.1%. The type of elution used was isocratic. The equipment was programmed with the following values: 20 min run time, 30 °C, 205 nm, and a flow rate of 0.800 mL/min. The aqueous extract of RGT was briefly injected, followed by the injection of each of the standards at five different concentrations (50, 25, 20, 15, 10, and 5 µg/mL). The results obtained were used for the construction of standard curves for the determination of the concentration at which each of the identified metabolites was found in 1 g RGT. For each concentration, 20 µL was injected. The syringe was flushed with copious amounts of methanol, acetonitrile, and water between each component change. The experiments were performed in triplicate from three independent experiments.

#### 4.2.3. UHPLC-ESI-QTOF-MS/MS Analysis

The aqueous extract part of RGT was analyzed using UHPLC-ESI-QTOF-MS/MS for the identification and quantification of target compounds. This technique combined high-resolution chromatographic separation (UHPLC) with high-resolution mass spectrometry (QTOF-MS/MS) and electrospray ionization (ESI) to achieve precise identification and sensitive quantification of compounds present in RGT. The analysis was performed in triplicate using the Zorbax Eclipse Plus C18 column at a temperature of 60 °C. The separation of components was achieved through gradient elution using 0.1% aqueous formic acid (A) and 0.1% formic acid in acetonitrile (B). The flow rate was set to 0.4 mL/min, with a 2 μL sample injected into the column. A linear gradient was employed as follows: 98% (A) for the first 10 min, 30% (A) by 50 min, 5% (A) at 51 min, and maintained at 5% (A) until 60 min, followed by a post-run phase of 5 min. The extraction solvent was used as a blank. The aqueous extract of RGT sample was prepared at a concentration of 2% *w*/*v*. The mass studies were achieved in positive mode, and the rest of the conditions for the LC-MS/MS analysis are listed in Table 3.

The raw data analysis was conducted in several stages: first, peak collection and deconvolution were carried out for each sample individually; next, data alignment was performed across the samples; finally, the data were integrated for each distinct and aligned sample. The data were then combined into a single matrix using Agilent software packages: Mass Hunter Qualitative Analysis (Agilent Mass Hunter Qualitative Analysis (Qual) B.07.00), Agilent Profinder (Agilent Mass Hunter Profinder B.06.00), and Agilent Mass Profiler Professional (Agilent Mass Profiler Professional (MPP) B.13.0). The identification of individual compounds was based on fragmentation patterns available in the validated metabolite mass tandem database (METLIN), supported by reports from the literature.

#### 4.2.4. Synthesis of Silver Nanoparticles Using Roasted Green Tea

The synthesis of nanoparticles was carried out using a 2% *w*/*v* aqueous extract of RGT at a pH of 10.5. The aqueous extract of RGT was heated to 60 °C. An equal volume (1:1) of a 20 mM silver nitrate solution was added, and the mixture was kept under constant stirring (650 rpm) and temperature for 60 min. The resulting solution underwent three centrifugation processes and two washing processes with Milli-Q water to remove any organic residues. Each centrifugation was performed for 20 min at 4000 rpm at a temperature of 4 °C, using a benchtop centrifuge equipped with a fixed-angle rotor designed for Falcon tubes.

#### 4.2.5. Characterization Techniques of Silver Nanoparticles

The optical property of AgNPs was evaluated using UV–visible spectrophotometry in the wavelength range of 200 to 1000 nm with a resolution of 1 nm. Morphology was determined through transmission electron microscopy (TEM, JEOL, JEM-1010 Tokyo, Japan) at an acceleration voltage of 80 kV. Sample preparation involved dispersing 40 µL of AgNPs in 1 mL of isopropanol, followed by depositing 5 μL onto carbon-coated copper grids and allowing solvent evaporation at room temperature. Size distribution of AgNPs was estimated from TEM micrographs using ImageJ software (NIH, Bethesda, MD, USA). AgNPs were analyzed using Fourier-transform infrared spectroscopy (FTIR, PerkinElmer™ Frontier Spectrometer—ATR mode, Waltham, MA, USA) with a resolution of 4 cm^−1^ in the 4000 to 400 cm^−1^ regions. X-ray diffraction patterns of the nanoparticles were correlated using crystallographic card number 040783.

#### 4.2.6. Preparation of Alginate–Gelatine Hydrogel with AgNPs

An amount of 100 mL of deionized water was heated to 60 °C. Once this temperature was reached, 4 g of alginate was added, the heating was stopped, and the mixture was covered with aluminum foil to prevent evaporation. It was stirred at 800 rpm for 1 h until a homogeneous mixture without lumps or bubbles was obtained. Then, 4 µg/mL of AgNPs (non-cytotoxic therapeutic dose) was slowly dispersed into the aqueous alginate solution. The mixture was stirred for 1 h at constant agitation (800 rpm) and room temperature. When the AgNPs were observed to be completely dispersed, the solution was heated to 50 °C, and finally, gelatin at 2.5 % *w*/*v* was added. The mixture was thoroughly combined using a metal spatula, poured into a Petri dish, and the hydrogel was left for 24 h to achieve maximum conformation. The hydrogels were characterized using FTIR (PerkinElmer™ Frontier Spectrometer—ATR mode, MA, USA) with a resolution of 4 cm^−1^ in the 4000 to 400 cm^−1^ regions and X-ray diffraction The X-ray diffraction analysis was conducted using a copper (Cu) anode X-ray diffractometer. During the measurement, an average wavelength of 1.54184 Å was utilized, corresponding to the Kα1 radiation. The equipment configuration included a voltage of 30 kV and a current of 10 mA. A “Locked Coupled” scanning mode was employed, starting at approximately 5° (4.99816°) and extending to a range that can reach up to 90° or more, with an increment of 0.0202536° per step. The total measurement time was approximately 1485.37 s (around 24.7 min). Additionally, the goniometer used had a diameter of 282 mm.

#### 4.2.7. SHEDs Culture

The protocol for isolating, culturing, and characterizing human exfoliated deciduous teeth cells (SHEDs) was approved by the bioethics committee of the ENES León Unit, UNAM, under the registration code CE_16 004_SN. This protocol adheres to national health regulations and the Helsinki Declaration, ensuring that informed consent was obtained prior to the extraction. Pulpal tissue from anterior deciduous teeth of patients aged 6–7 years, free from caries, was used. The tissue was handled inside a horizontal laminar flow hood. The isolated explants, measuring 1 × 1 mm, were incubated in minimum essential medium (MEM, Sigma-Aldrich) supplemented with 20% fetal bovine serum (FBS, Gibco), 1% glutamine (Gibco), and 1% antibiotic (PenStrep, Sigma-Aldrich). After thawing cryopreserved cells in an incubator for 5–10 min, they were transferred to 10 cm culture plates containing MEM culture medium with 10% fetal bovine serum (FBS), 1% glutamine, and 1% antibiotics. Cells were incubated at 37 °C with 5% CO_2_, and the medium was replaced every two days until the cells reached over 80% confluence.

#### 4.2.8. SHEDs Characterization

The SHEDs displayed a fibroblast-like morphology and achieved 90% confluence. After four population doublings (PDL 4), the cells were characterized based on their plastic adherence and fibroblastic morphology. Flow cytometry was employed to analyze the expression of CD90 (Monoclonal Anti-CD90-PE produced in mouse, SAB470086) and CD105 (Monoclonal Anti-CD105-PE produced in mouse, SAB4700261) utilizing antibodies from Sigma-Aldrich.

#### 4.2.9. AgNPs-SHEDs Cytotoxicity Dose–Response

For the cell viability experiments, the AgNPs solution at 20 mM was diluted in a series of 12 successive 1:1 dilution. The highest concentration was 20 mM (equivalent to 2157.4 µg/mL of AgNPs) and the lowest was 1.05 µg/mL. The SHEDs were subcultured in a 96-well microplate and washed with PBS, followed by detachment using 0.25% trypsin-0.025% EDTA-2Na solution. The cell count was determined using a hemocytometer with trypan blue exclusion under light microscopy. A total of 1 × 10^5^ cells/mL was seeded and allowed to attach for 48 h. The cells were then treated with AgNPs at concentrations ranging from 0 to 2157.4 µg/mL (*n* = 9) and incubated for 24 h with fresh culture medium. Cell viability was assessed using the MTT assay, where cells were incubated with 0.2 mg/mL MTT in MEM containing 10% FBS for 7 h. The resulting formazan crystals were dissolved in 0.1 mL of dimethyl sulfoxide, and absorbance was measured at 570 nm using a microplate spectrophotometer. The cytotoxicity was based on the ISO 10993-5:2009 Biological evaluation of medical devices—Tests for in vitro cytotoxicity [96].

#### 4.2.10. Indirect Cytotoxicity Evaluation of Alg-Gel Hydrogels Containing AgNPs

The indirect cytotoxicity assay of the Alg-Gel hydrogels containing AgNPs and without them was performed according to the ISO 10993-5:2009 Biological evaluation of medical devices—Tests for in vitro cytotoxicity. Cells were cultured as mentioned above. The Alg-Gel and Alg-Gel-AgNPs hydrogels were immersed in 4 mL of MEM, 10% FBS, 1% antibiotics, and left for 24 h under agitation. After incubation, the cell medium was replaced with extraction medium (medium containing Alg-Gel-AgNPs and Alg-Gel) and further incubated for 24 h, 37 °C. Cell viability for each extraction medium was assessed using the MTT assay, with cells cultured in fresh MEM serving as the control. After removing the culture medium, 100 µL of MTT solution (0.2 mg/mL) was added to each well and incubated at 37 °C for 6 h. The solution was then aspirated, and 100 µL of DMSO was added to dissolve the formazan crystals. Absorbance was measured at 570 nm to determine viability, which was expressed as a percentage of viable cells.

#### 4.2.11. Evaluation of the Hydrogel for Osteoblastic Differentiation

To induce osteoblastic differentiation, SHEDs were cultured in an osteogenic differentiation medium containing MEM, 10% FBS, 1% antibiotics, 0.1 mM dexamethasone, 5 mM β-glycerophosphate, 50 µg/mL ascorbic acid, 20 ng/mL transforming growth factor-β3, and 5 ng/mL fibroblast growth factor-2. Standard subcultured cells served as controls. Mineralization was evaluated using Alizarin Red staining, followed by dissolving the stain in a 5% 2-isopropanol and 10% acetic acid solution for 16 h. The absorbance of the dissolved Alizarin Red was measured at 550 nm using a spectrophotometer to quantify the calcified mineral deposition.

#### 4.2.12. Statistical Analysis

All measurements were conducted in triplicate, and the results are expressed as mean ± standard deviation (SD) from at least three independent experiments. Statistical significance was determined using one-way analysis of variance (ANOVA) with a threshold of *p* < 0.05, analyzed through Prisma 10 for Windows 64-bit, version 10.3.1.

## Figures and Tables

**Table 1:** Concentrations (mg/L) of each identified compound in 1 g of RGT.

Peak	Compound	Concentration [mg/L]
1	Gallic acid	86.09
2	Gallocatechin	141.90
3	Epigallocatechin	58.34
4	Catechin	33.38
5	Caffeine	312.77
6	Epigallocatechin gallate	81.31
7	Epicatechin	29.82

**Table 2:** Mass spectrometry profile of chemical compounds in RGT.

*m*/*z*	Z	Abundance	Name	Formula	Ion
170.0204	1	1118.6	Gallic acid	C_7_H_6_O_5_	(M+H)+
306.0720	1	146,324.05	(−)-Gallocatechin	C_15_H_14_O_7_	(M+H)+
458.0839	1	39,087.56	(−)-Epigallocatechin (−)-Epigallocatechin gallate	* C_22_H_18_O_11_	(M+H)+
290.07	1	58,827.5	(−)-Catechin (−)-Epicatechin	** C_15_H_14_O_6_	(M+H)+
194.19	1	1,096,381	Caffeine	C_8_H_10_O_2_	(M+H)+

*m*/*z*: Mass-to-charge ratio in Daltons (Da). Z: Ion charge. Abundance represents relative abundance in arbitrary units. Ion (M+H): Signifies the molecular ion plus the hydrogen ion. ** Isomers. * Enantiomers.

**Table 3:** Mass Spectrometry (MS) and MS/MS conditions (Agilent 6545 QTOF System).

Ionic Mode	Dual AJS ESI	Minimum MS (*m*/*z*) range	100
Minimum MS Range (*m*/*z*)	Positive	Maximum MS (*m*/*z*) range	1000
Ion Polarity	Dual AJS ESI	MS Scan Rate Acquisition Rate (spectra/second)	3
Maximum MS Range (*m*/*z*)	300 °C	Minimum MS/MS (*m*/*z*) range	50
Source	300 °C	Maximum MS (*m*/*z*) range	1000
AJS ESI MS Scan Rate	10 min/L	MS/MS Scan Rate (acquisition rate) (spectra/second)	2
Gas Temperature	45 psig	Data Storage Mode	Medium (−4 amu)
Minimum MS/MS Range (*m*/*z*)	3500 V	MS/MS Relative Threshold (%)	Both
Envelop Gas Temperature	500	Absolute Limit (Threshold Abs)	0.010
Maximum MS Range (*m*/*z*)	120 V	Relative Limit (Threshold rel) %	200
Gas Flow	(Positive) 121.05087300; 922.00979800	Instrument Model	0.010
MS/MS Scan Rate	Medio (−4 *m*/*z*)	Component Model	G6545A
Nebulizer Pressure	15–30 eV	MS Relative Threshold (%)	0.010
Data Storage Mode	200	Limit absolute de MS/MS (MS/MS Abs. Threshold)	5

The conditions used for LC-MS/MS analysis on the Agilent 6545 QTOF system are described. In this analysis, the Dual AJS ESI ionization mode is employed in positive polarity. The detectable mass range varies from 100 to 1000 *m*/*z*, with a mass spectra acquisition rate of 3 per second and an MS/MS spectra acquisition rate of 2 per second. Gas temperatures of 300 °C were used for both the source and the collision gas. The gas flow rate was 10 min per liter, with a nebulizer pressure of 45 psig. Capillary and nozzle voltages of 3500 V and 500 were applied, respectively. The collision energy was set in the range of 15–30 eV, while the MS/MS isolation width was established at medium (−4 *m*/*z*). Additionally, reference masses were provided for positive ions, and a G6545A instrument model was used. The absolute and relative limits for mass and MS/MS detection were 0.010 and 200, respectively.

## Data Availability

The data presented in this study are available on request from the corresponding authors.
